# TAVI in patients with low-flow low-gradient aortic stenosis–short-term and long-term outcomes

**DOI:** 10.1007/s00392-022-02011-4

**Published:** 2022-03-23

**Authors:** Julius Steffen, Nikolas Reißig, David Andreae, Markus Beckmann, Magda Haum, Julius Fischer, Hans Theiss, Daniel Braun, Martin Orban, Konstantinos Rizas, Sebastian Sadoni, Michael Näbauer, Sven Peterss, Jörg Hausleiter, Steffen Massberg, Simon Deseive

**Affiliations:** 1 Medizinische Klinik und Poliklinik I, LMU Klinikum, LMU München, Marchioninistr. 15, 81377 Munich, Germany; 2 German Centre for Cardiovascular Research (DZHK), Partner site Munich, Munich, Germany; 3 Herzchirurgische Klinik und Poliklinik, LMU Klinikum, LMU München, Marchioninistr. 15, 81377 Munich, Germany

**Keywords:** Low-flow low-gradient, Aortic stenosis, TAVI, VARC-3

## Abstract

**Objectives:**

The study objective was to characterize different groups of low-flow low-gradient (LFLG) aortic stenosis (AS) and determine short-term outcomes and long-term mortality according to Valve Academic Research Consortium-3 (VARC-3) endpoint definitions.

**Background:**

Characteristics and outcomes of patients with LFLG AS undergoing transcatheter aortic valve implantation (TAVI) are poorly understood.

**Methods:**

All patients undergoing TAVI at our center between 2013 and 2019 were screened. Patients were divided into three groups according to mean pressure gradient (dPmean), ejection fraction (LVEF), and stroke volume index (SVi): high gradient (HG) AS (dPmean ≥ 40 mmHg), classical LFLG (cLFLG) AS (dPmean < 40 mmHg, LVEF < 50%), and paradoxical LFLG (pLFLG) AS (dPmean < 40 mmHg, LVEF ≥ 50%, SVi ≤ 35 ml/m^2^).

**Results:**

We included 1776 patients (956 HG, 447 cLFLG, and 373 pLFLG patients). Most baseline characteristics differed significantly. Median Society of Thoracic Surgeons (STS) score was highest in cLFLG, followed by pLFLG and HG patients (5.0, 3.9 and 3.0, respectively, *p* < 0.01). Compared to HG patients, odds ratios for the short-term VARC-3 composite endpoints, technical failure (cLFLG, 0.76 [95% confidence interval, 0.40–1.36], pLFLG, 1.37 [0.79–2.31]) and device failure (cLFLG, 1.06 [0.74–1.49], pLFLG, 0.97 [0.66–1.41]) were similar, without relevant differences within LFLG patients. NYHA classes improved equally in all groups. Compared to HG, LFLG patients had a higher 3-year all-cause mortality (STS score-adjusted hazard ratios, cLFLG 2.16 [1.77–2.64], pLFLG 1.53 [1.22–193]), as well as cardiovascular mortality (cLFLG, 2.88 [2.15–3.84], pLFLG, 2.08 [1.50–2.87]).

**Conclusions:**

While 3-year mortality remains high after TAVI in LFLG compared to HG patients, symptoms improve in all subsets after TAVI.

**Graphical abstract:**

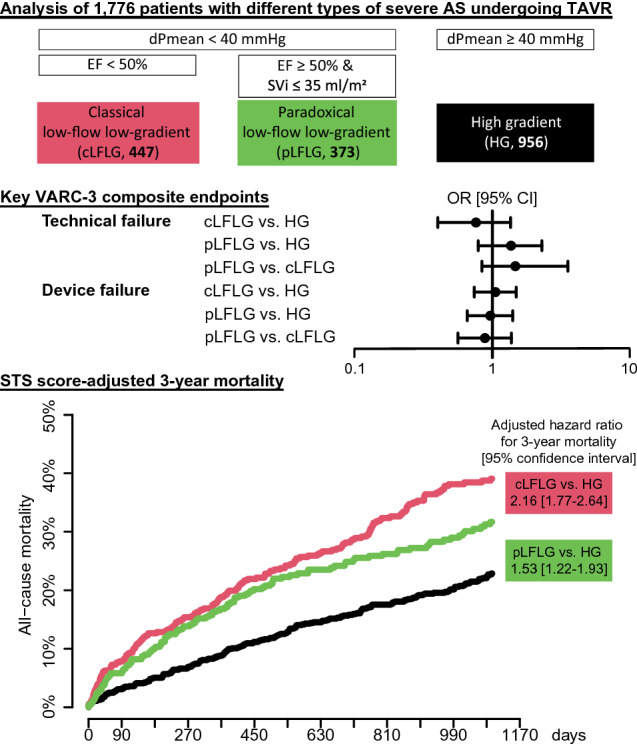

**Supplementary Information:**

The online version contains supplementary material available at 10.1007/s00392-022-02011-4.

## Introduction

Transcatheter aortic valve implantation (TAVI) is the standard of care for most elderly patients with severe symptomatic aortic valve stenosis (AS) [1]. Its ability to prolong survival and diminish symptoms with acceptable complication rates has been shown in many studies [2, 3].

While high-gradient (HG) AS is the classical indication in most TAVI patients, some patients undergoing TAVI present with low gradient AS. Low gradient AS is diagnosed when calculated aortic valve area (AVA) is < 1 cm^2^ and mean pressure gradients (dPmean) are < 40 mmHg [4]. Patients are further divided into a classical low-flow low gradient (LFLG) AS when left ventricular ejection fraction (LVEF) is < 50%, and paradoxical LFLG AS when LVEF is normal but stroke volume index (SVi) is reduced [5]. LFLG patients often suffer from many relevant comorbidities such as atrial fibrillation (AF), mitral or tricuspid regurgitation (MR, TR), or severe coronary artery disease (CAD), and frequently show heart failure symptoms that go beyond the typical symptoms of aortic stenosis. Patients with classical LFLG AS are essentially heart failure patients with reduced LVEF (HFrEF). In contrast, paradoxical LFLG patients are thought to have high afterload and impaired filling due to remodeling and consecutive low-flow state despite preserved systolic function [6].

Diagnosis of LFLG AS is difficult because stenosis severity can be underestimated by dPmean as this is subject to flow velocity and overestimated by AVA because of impeded valve opening due to low flow in the outflow tract. Therefore, multimodality imaging is necessary and aortic valve replacement should be recommended after careful consideration [1, 7].

Unlike for classical high gradient AS, the short-term clinical success rates and long-term outcomes of LFLG AS patients after TAVI have not been evaluated in larger patient cohorts. Subanalyses of randomized multicenter studies [8, 9] or single-center studies [10] indicate that long-term clinical success may be limited in LFLG patients compared to patients with HG stenoses. However, robust outcome data concerning the different subtypes of LFLG are still missing. Therefore, the objective of this analysis was to (i) characterize LFLG patients in a large cohort, (ii) compare short-term outcomes according to the newly published Valve Academic Research Consortium (VARC-3) guidelines [11], and (iii) analyze long-term mortality and prognostic factors.

## Methods

### Study population

**Fig. 1:**
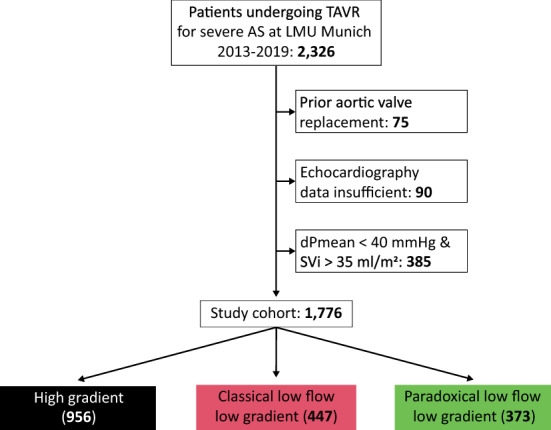
Study flow chart All patients undergoing transcatheter aortic valve replacement for severe AS between 2013 and 2019 were screened. Patients with prior aortic valve replacement and patients with insufficient echocardiography data were excluded. Patients were divided into groups according to aortic valve flow patterns. Patients with normal-flow low-gradient AS were not included in the analysis. *dPmean* transvalvular pressure gradient, *SVi* stroke volume index, *TAVI* transcatheter aortic valve implantation

All patients undergoing transfemoral TAVI for severe AS at LMU University Hospital Munich between 2013 and 2019 were retrospectively evaluated for this analysis (Fig. 1). Patients with missing data on echocardiography and patients with prior aortic valve replacement were excluded from the analysis. In line with the guidelines, patients with dPmean < 40 mmHg, preserved LVEF and normal SVi were not included since the presence of severe AS is the subject of debate in these patients [1].

The appropriateness of TAVI in all patients was evaluated by an interdisciplinary heart team, consisting of cardiac surgeons and interventional cardiologists as recommended by the guidelines [1].

Clinical and procedural data of all patients were collected as part of our routine documentation according to local quality control requirements and the EVERY-Valve registry. This study was approved by the local ethics committee. Follow-up was performed mainly by telephone calls and visits at our outpatients’ clinic after 30 days and yearly thereafter [12]. The cause of death was determined by inquiring treating physicians and general practitioners involved in the care of the patients.

### Echocardiographic definition of 3 groups

Echocardiography was part of the routine pre-interventional work-up of patients, evaluating valve and chamber morphology and function according to current guidelines [4]. All pre-interventional echocardiography images have been reassessed by an independent echocardiographer. Aortic valve area (AVA) was evaluated using the continuity equation method. Continuous wave and pulsed wave doppler echocardiograms were used to calculate stroke volumes. Patients were divided into three groups according to dPmean, LVEF, and SVi[5]: Patients with a dPmean ≥ 40 mmHg have HG AS (i), patients with a dPmean < 40 mmHg were split into (ii) classical LFLG (LVEF < 50%) (cLFLG) and (iii) paradoxical LFLG (LVEF ≥ 50% and SVi ≤ 35 ml/m^2^) (pLFLG).

### TAVI procedure and medication

All patients underwent transfemoral access and local anesthesia for the TAVI procedure. The choice of prosthesis as well as the performance of pre- or post-dilation was left to the interventionalist’s discretion. For peri-procedural anticoagulation, unfractionated heparin was used (50–70 IU/kg body weight). Suture-mediated closure devices were used for access-site closure. Oral anticoagulation was continued after the procedure in patients with indications for such. All other patients were treated with 100 mg acetylsalicylic acid plus 75 mg clopidogrel for three months followed by lifelong 100 mg acetylsalicylic acid. If patients had also undergone percutaneous coronary intervention (PCI) and had an indication for oral anticoagulation, triple regimens were conducted according to the guidelines [13].

### Clinical endpoints

The primary endpoints were defined according to the recently published VARC-3 guidelines [11] and included the composite endpoints (i) technical failure at the end of the procedure, and (ii) device failure at 30 days, as well as (iii) 3-year all-cause mortality. Secondary endpoints included the components of the VARC-3 composite endpoints, such as procedural death, structural vascular or cardiac complications, conversion to open surgery, prosthesis dislocation, the use of a second valve prosthesis, immediate and late (30 days) vascular intervention or surgery, 30-day mortality, elevated pressure gradients, relevant paravalvular regurgitation (PVR) on echocardiography, stroke, relevant bleeding (BARC type 3) [14], stage 3 or 4 acute kidney injury (AKI) according to KDIGO [11], permanent pacemaker implantation, and myocardial infarction at 48 h (early MI). PVR after TAVI was assessed according to the scale proposed by Pibarot and colleagues [15].

### Statistical analysis

Continuous variables are presented as median [interquartile range, IQR]. Shapiro–Wilk test was used for normality assessment. Categorial variables are presented as absolute numbers and percentages. Values from different groups were compared using Fisher’s exact test, Chi-squared test, Kruskal–Wallis test, or Wilcoxon test as appropriate. Odds ratios were calculated to compare outcomes of the three groups. The two LFLG groups were compared to HG in combination as well as separately and against each other. Survival was compared using the Kaplan–Meier estimation and log-rank test. For the analysis of cardiovascular death, a competing risk model was used [16]. Cox regression was used to adjust groups for the Society of Thoracic Surgeons Score (STS score). A *p* value of < 0.05 was considered statistically significant. All statistical analysis was performed with R, version 4.0.0 (RStudio Inc., Boston, MA, USA), graphs were designed with Prism 9 for macOS, version 9.1.1 (GraphPad Software, San Diego, CA, USA) and Adobe Illustrator version 24.0.3 (Adobe Inc., San Jose, CA, USA).

## Results

### Clinical characteristics at baseline

A total of 1,776 patients between 2013 and 2019 were included in the study, divided into 956 patients with HG (54%), 447 patients with cLFLG (25%) and 373 patients with pLFLG (21%) aortic stenosis (Fig. 1). Overall median follow-up time was 2.6 [1.3–3.7] years, with a completeness of 1-year and 3-year follow-up data of 98 and 74%, respectively. Patient baseline characteristics are presented in Table 1. Differences between groups were found in most variables. Patients in the HG group had the lowest median STS-scores and generally lower rates of comorbidities. Patients with cLFLG AS were mainly male (69.6%), suffered more frequently from coronary artery disease or chronic kidney disease (CKD), and showed the highest median STS-scores. Most pLFLG-patients were female and showed the highest rate of atrial fibrillation.

**Table 1:** Patient characteristics

	HG (*N* = 956)	cLFLG (*N* = 447)	pLFLG (*N* = 373)	Total (*N* = 1776)	*p* value
Male sex	440 (46.0%)	311 (69.6%)	166 (44.5%)	917 (51.6%)	**< 0.01**
Age (years)	81.2 [77.3–85.4]	82.0 [77.4–86.3]	82.2 [78.3–85.6]	81.7 [77.5–85.7]	0.19
Body mass index (kg/m^2^)	25.8 [23.4–29.4]	25.5 [22.7–28.8]	26.0 [23.8–28.7]	25.8 [23.4–29.1]	0.19
Body surface area (m^2^)	1.8 [1.7–2.0]	1.9 [1.7–2.0]	1.8 [1.7–2.0]	1.8 [1.7–2.0]	**0.02**
STS-score	3.0 [2.0–5.0]	5.0 [3.0–7.3]	3.9 [2.2–6.0]	3.8 [2.1–6.0]	**< 0.01**
Diabetes mellitus type 2	249 (28.3%)	148 (35.3%)	118 (33.8%)	515 (31.2%)	**0.02**
Hypertension	785 (88.7%)	392 (92.9%)	324 (92.8%)	1501 (90.6%)	**0.01**
Smoker (active or past)	174 (19.7%)	103 (24.5%)	54 (15.4%)	331 (20.0%)	**< 0.01**
Hypercholesterolemia	351 (40.3%)	170 (41.3%)	161 (46.4%)	682 (41.8%)	0.14
Positive family history	86 (9.0%)	35 (7.8%)	46 (12.3%)	167 (9.4%)	0.07
Chronic kidney disease	367 (38.4%)	279 (62.4%)	178 (47.7%)	824 (46.4%)	**< 0.01**
Atrial fibrillation	193 (20.2%)	163 (36.5%)	155 (41.6%)	511 (28.8%)	**< 0.01**
Coronary artery disease	500 (56.2%)	314 (73.5%)	222 (63.4%)	1036 (62.2%)	**< 0.01**
Prior MI	107 (11.2%)	119 (26.6%)	50 (13.4%)	276 (15.5%)	**< 0.01**
Prior PCI	258 (29.3%)	195 (46.2%)	113 (32.3%)	566 (34.2%)	**< 0.01**

Data are presented as *n* (%) or median [IQR]. A *p* value of < 0.05 was considered significant

*STS-score* society of thoracic surgeons score, *MI* myocardial infarction, *PCI* percutaneous coronary intervention

Baseline echocardiographic characteristics differed between groups in accordance with the criteria described above and are shown in Table 2. Patients in the HG group had the lowest rate of more than mild TR, and cLFLG patients had a higher rate of relevant MR and right-ventricular/right-atrial pressure gradients. Computer-tomographic data are shown in Supplemental Table 1.

**Table 2:** Echocardiographic characteristics

	HG (*N* = 956)	cLFLG (*N* = 447)	pLFLG (*N* = 373)	Total (*N* = 1776)	*p* value
AVA (cm^2^)	0.7 [0.5–0.8]	0.7 [0.6–0.9]	0.7 [0.6–0.8]	0.7 [0.6–0.8]	**< 0.01**
AVAi (cm^2^/m^2^)	0.4 [0.3–0.4]	0.4 [0.3–0.5]	0.4 [0.3–0.4]	0.4 [0.3–0.4]	**< 0.01**
Maximum gradient (mmHg)	76.0 [68.0–89.0]	43.0 [33.0–52.0]	48.0 [39.0–56.0]	64.0 [46.0–78.0]	**< 0.01**
Mean gradient (mmHg)	48.0 [43.0–56.0]	26.0 [20.0–32.0]	28.0 [23.0–35.0]	40.0 [28.0–49.0]	**< 0.01**
SV (ml)	68.0 [57.0–83.0]	51.0 [40.0–59.0]	55.0 [46.0–60.0]	59.0 [49.0–72.0]	**< 0.01**
SVi (ml/m^2^)	37.9 [31.5–44.9]	27.3 [21.8–32.7]	29.5 [26.0–32.4]	32.4 [27.0–39.2]	**< 0.01**
LVEF (%)	55.0 [50.0–58.0]	40.0 [35.0–45.0]	55.0 [53.0–56.0]	55.0 [45.0–55.0]	** < 0.01**
TAPSE (mm)	22.0 [18.0–25.0]	17.0 [14.0–20.0]	20.0 [16.0–23.0]	20.0 [17.0–23.0]	**< 0.01**
AR grade 2	96 (10.0%)	57 (12.8%)	26 (7.0%)	179 (10.1%)	**0.02**
MR 3–4/4	37 (3.9%)	41 (9.2%)	15 (4.0%)	93 (5.3%)	**< 0.01**
TR 2–3/3	82 (9.1%)	80 (19.1%)	74 (20.2%)	236 (14.0%)	**< 0.01**
RV/RA-gradient (mmHg)	34.5 [26.0–45.0]	38.0 [29.0–48.0]	35.0 [27.0–42.0]	36.0 [27.0–45.0]	**< 0.01**
E (cm/s)	119.0 [106.0–136.0]	117.0 [103.0–132.2]	115.0 [108.0–133.0]	118.0 [106.0–135.0]	0.67
A (cm/s)	119.0 [107.0–137.0]	104.5 [81.2–119.8]	113.0 [101.5–128.5]	116.0 [103.8–133.2]	**< 0.01**
Septal E’ (cm/s)	5.1 [4.1–6.1]	4.9 [4.2–6.0]	5.9 [4.7–7.4]	5.2 [4.2–6.4]	**< 0.01**
Lateral E’ (cm/s)	7.0 [5.4–8.6]	7.3 [5.9–9.9]	8.6 [7.2–10.7]	7.4 [5.8–9.4]	**< 0.01**
LA volume (ml)	79.8 [60.7–98.1]	93.8 [76.1–117.0]	87.9 [60.3–111.8]	84.0 [63.0–107.0]	**< 0.01**
LVIDd (mm)	4.6 [4.1–5.1]	5.2 [4.5–5.7]	4.5 [4.0–5.0]	4.7 [4.2–5.3]	**< 0.01**
IVSd (mm)	1.4 [1.2–1.5]	1.2 [1.1–1.4]	1.3 [1.1–1.5]	1.3 [1.2–1.5]	**< 0.01**
LVPWd (mm)	1.2 [1.1–1.4]	1.1 [1.0–1.3]	1.1 [1.0–1.3]	1.2 [1.0–1.3]	**< 0.01**

Data are presented as *n* (%) or median [IQR]. A *p* value of < 0.05 was considered significant

*AVA* aortic valve area, *AVAi* aortic valve area index, *dPmax* maximum pressure gradient, *dPmean* mean pressure gradient, *SV* stroke volume, *SVi* stroke volume index, *LVEF* left-ventricular ejection fraction, *TAPSE* tricuspid annular plane systolic excursion, *AR* aortic regurgitation, *MR* mitral regurgitation, *TR* tricuspid regurgitation, *RV/RA gradient* right ventricular-right atrial pressure gradient, *LA* left atrium, *LVIDd* left ventricular internal diastolic diameter, *IVSd* interventricular septum diastolic diameter, *LVPWd* left ventricular posterior wall diastolic diameter

Concerning the procedure, larger valve prostheses were used in cLFLG compared to HG or pLFLG, while prosthesis types were similar between the groups. A concomitant PCI was performed in 18% of the cLFLG patients, which was significantly more often than in the other groups (Supplemental Table 1).

### Technical and clinical outcomes

**Fig. 2:**
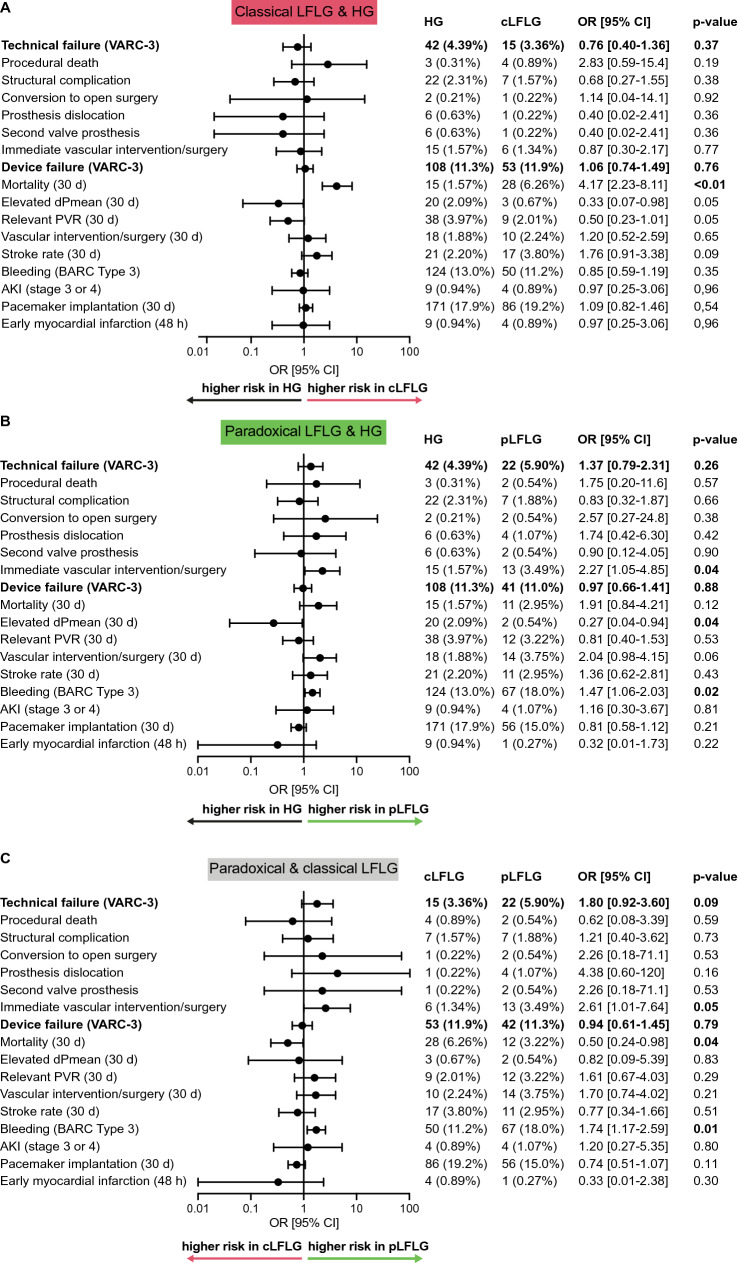
Technical and clinical outcomes Short-term outcomes (up to 30 days) for the two LFLG groups were compared to HG and compared to each other according to Valve Academic Research Consortium-3 (VARC-3) endpoints. The composite endpoints of a technical failure (consisting of procedural death, structural cardiac complications, conversion to open surgery, prosthesis dislocation, the use of a second valve prosthesis, or immediate vascular intervention or surgery) or device failure at 30 days (consisting of the composite endpoint technical failure, 30-day mortality, elevated pressure gradients or relevant paravalvular regurgitation on echocardiography, or vascular surgery/intervention at 30 days, stroke, relevant bleeding, acute kidney injury (AKI) and permanent pacemaker implantation) occurred at similar frequencies. However, there were differences in single components. **a** The 30-day mortality was significantly higher in cLFLG compared to HG. **b** For pLFLG patients, the risk of relevant bleeding or the necessity for vascular interventions were significantly increased in comparison to HG patients. **c** While pLFLG patients had a higher risk for bleeding and vascular intervention/surgery, cLFLG patients had a higher 30-day mortality. *OR* denotes odds ratio. *AKI* acute kidney injury, *cLFLG* classical low-flow low-gradient, *dPmean*, mean transvalvular pressure gradients, *HG* high gradient, *pLFLG* paradoxical low-flow low-gradient, *VARC-3* valve academic research consortium-3

Short-term technical and clinical outcomes for each group were analyzed according to VARC-3 criteria. The first primary endpoint (VARC-3 composite endpoint technical failure) occurred at similar rates in the LFLG groups combined (Supplemental Fig. 1) as well as separately (Fig. 2a, b) compared to the HG group. The odds ratios (OR) for technical failure were 0.76 [0.40–1.36] (*p* = 0.37) in classical LFLG compared to HG patients and 1.37 [0.79–2.31] (*p* = 0.26) in paradoxical LFLG compared to HG. The OR for pLFLG compared to cLFLG was 1.80 [0.92–3.60] (*p* = 0.09, Fig. 2c).

The second primary endpoint, the VARC-3 composite endpoint of device failure at 30 days, was observed at similar rates in the LFLG groups compared to the HG patients.

Notably, the 30-day mortality was generally higher in the two LFLG groups together (4.9%) (Supplemental Fig. 1) compared to HG (1.6%, *p* < 0.01, driven by a higher 30-day mortality in cLFLG patients (6.3%) compared to pLFLG patients (3.0%) (*p* = 0.04). In the pLFLG group, an increased risk for relevant (BARC Type 3) bleeding was observed (Fig. 2).

**Fig. 3:**
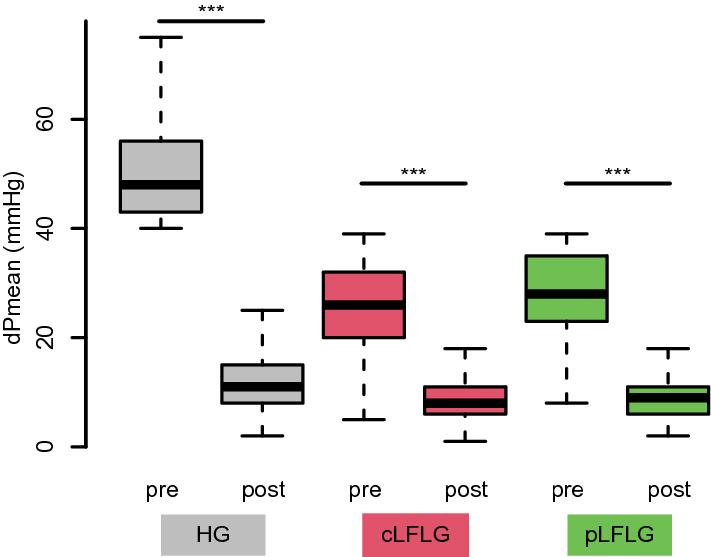
Mean transvalvular pressure gradients before and after TAVI The graph depicts the mean transvalvular pressure gradients before TAVI and at follow-up for each group. Mean pressure gradients were 48 [43–56] mmHg before and 11 [8–15] mmHg after TAVI for HG, 26 [20–32] mmHg before and 8 [6–11] mmHg after TAVI for cLFLG, and 28 [23–35] mmHg before and 9 [6–12] mmHg after TAVI for pLFLG. There was a significant reduction of dPmean after TAVI in all three groups (*p* < 0.01 for all). *cLFLG* classical low-flow low-gradient, *dPmean* mean transvalvular pressure gradients**,** *HG* high gradient, *pLFLG* paradoxical low-flow low-gradient, *TAVI* transcatheter aortic valve implantation

**Fig. 4:**
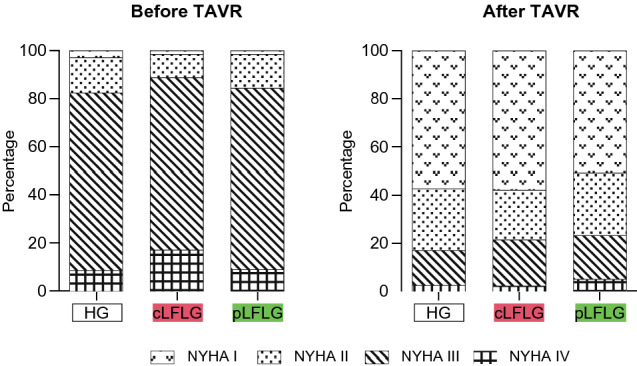
NYHA Class Outcomes Patients’ New York Heart Association (NYHA) class was assessed before and at the latest possible follow-up up to three years after TAVI. Colors indicate NYHA class per group. There was a reduction of at least one NYHA class after TAVI in 76.3% of all patients without a relevant between-group difference. *cLFLG* classical low-flow low-gradient, *HG* high gradient, *NYHA* New York heart association, *pLFLG* paradoxical low-flow low-gradient, *TAVI* transcatheter aortic valve implantation

At echocardiographic follow-up, there was a significant reduction of dPmean after TAVI in all three groups (Fig. 3). Subjective outcomes in terms of the New York Heart Association (NYHA) class were collected during follow-up and were compared to baseline data. Most patients were NYHA class III or IV at baseline and NYHA class I at follow-up. There was a similarly strong improvement of at least one NYHA class in all three groups, with 76.9, 78.4, and 72.2% for HG, cLFLG, and pLFLG patients, respectively (*p* = 0.32) (Fig. 4).

### Mortality

**Fig. 5:**
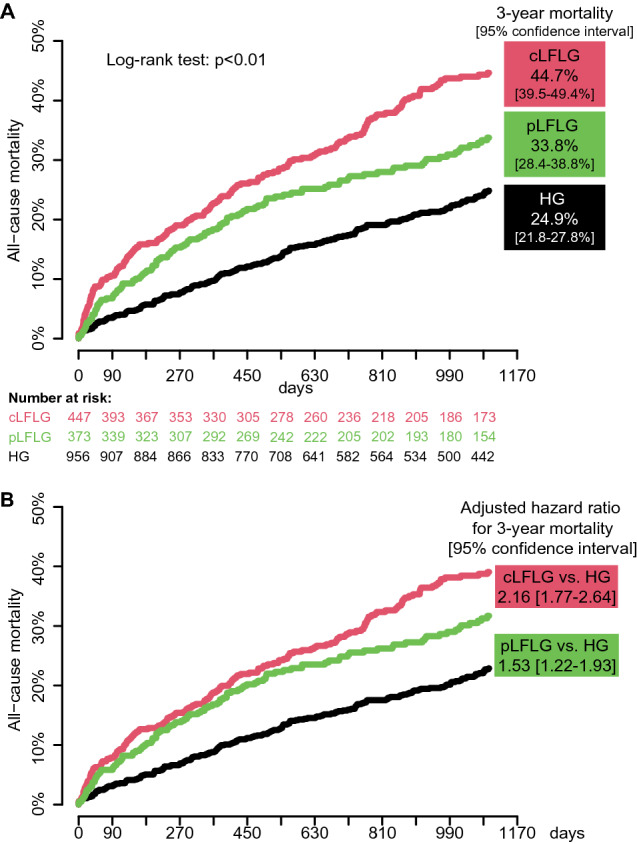
Estimated mortality curves in the overall study population Kaplan–Meier curves depicting 3-year mortality after TAVI. **a** Estimated mortality rates were significantly different between groups at 1 year, 2 years and at 3 years. **b** Mortality curves were adjusted to the median STS-score (3.78). Adjusted hazard ratios (HR) showed significantly increased estimated mortality rates for classical and paradoxical LFLG compared to HG patients. *cLFLG* classical low-flow low-gradient, *HG* high gradient, *pLFLG*, paradoxical low-flow low-gradient, *TAVI* transcatheter aortic valve implantation

All-cause mortality rates at 3 years were 24.9% [95% confidence interval (CI) 21.8–27.8%] for HG, 33.8% [95% CI 28.4–38.8%] for pLFLG, and 44.7% [95% CI 39.5–49.4%] for cLFLG, and differed significantly between all groups (log-rank test, *p* < 0.01, Fig. 5a). A comparison of the combined LFLG groups to HG is shown in Supplemental Fig. 2. The 3-year all-cause mortality was lower among pLFLG compared to cLFLG patients (HR 0.71 [95% CI 0.56–0.90], *p* < 0.01).

STS score-adjusted HR for 3-year all-cause mortality were 2.16 [95% CI, 1.77–2.64] for cLFLG and 1.53 [95% CI 1.22–1.93] for pLFLG compared to HG patients (Fig. 5b). There was a trend towards a lower STS score-adjusted mortality in paradoxical compared to classical LFLG patients (adjusted HR 0.80 [95% CI 0.63–1.02]). Adjusted HRs also indicated significantly increased mortality for cLFLG and pLFLG patients at 1 and 2 years.

**Fig. 6:**
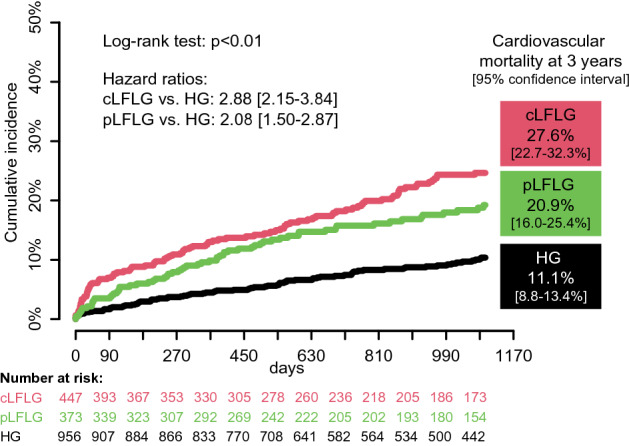
Estimated cardiovascular mortality at 3 years The cause of death was compared between the three groups in a competing risk analysis regarding cardiovascular mortality, which was more common in cLFLG and pLFLG patients. *cLFLG* classical low-flow low-gradient, *HG* high gradient, *pLFLG* paradoxical low-flow low-gradient, *TAVI* transcatheter aortic valve implantation

An additional competing risks analysis revealed that the cumulative incidence of cardiovascular death was significantly different between the three groups. While cardiovascular death was significantly more frequent among cLFLG (55.6%) and pLFLG (56.4%) compared to HG patients (46.0%, Chi-squared test *p* = 0.04), HG patients died from cancer more frequently (3.9 vs. 2.1 vs. 10.5%, Chi-squared test *p* < 0.01). The HR for cardiovascular death after 3 years was 2.88 [95% CI 2.15–3.84] for classical LFLG vs. HG and 2.08 [95% CI 1.50–2.87] for paradoxical LFLG vs. HG patients (Fig. 6 and Supplemental Table 2). The HR for cardiovascular death after 3 years for the combination of the two LFLG groups compared to HG was 2.47 [95% CI 1.90–3.21]. Within the LFLG groups, there was a trend to lower cardiovascular mortality among paradoxical compared to classical LFLG (HR 0.73 [95% CI 0.54–1.01], *p* = 0.06).

### Predictors of mortality

Hazard ratios from a univariate analysis of the impact of baseline characteristics on 3-year mortality rates for each group alone are compared in Supplemental Table 3. Age, AF, CKD, an elevated RV/RA gradient as well as the STS score are shown to affect mortality in the univariate analyses of all three groups.

In a second analysis incorporating all three groups together, type of AS, sex, age, body mass index, CKD, AF, CAD, prior MI and PCI, TAPSE, right-ventricular/right-atrial pressure gradient and mitral and tricuspid regurgitation were significant predictors for 3-year mortality in the univariate analysis. In the multivariate analysis, cLFLG prevailed as an independent mortality predictor while pLFLG did not (Supplemental Table 4).

## Discussion

In this retrospective analysis of 1,776 patients undergoing TAVI, we compared patients with LFLG AS to patients with HG AS. We found (i) different baseline characteristics, (ii) similar short-term complication rates according to VARC-3, (iii) comparable clinical success rates, but (iv) striking differences in long-term mortality.

This study is one of the largest published single-center analyses with an adequate follow-up time comparing outcomes of 447 patients with classical and 373 patients with paradoxical LFLG AS to patients with HG AS. It comprises an STS score-adjusted mortality analysis, a comparison of cardiovascular death rates, and one of the first to analyze LFLG patients according to the new VARC-3 endpoint definitions [17]. The fraction of cLFLG (25%) and pLFLG (21%) patients is substantially higher than in the published literature [5, 18]. Our center is a quaternary care hospital in Germany, offering all aspects of modern medicine with a selected patient cohort suffering from multiple grave comorbidities. Nevertheless, the high fraction of LFLG patients in this real-world setting underlines the importance of research in this specific cohort of patients.

### Key baseline characteristics resemble three different entities

This study confirmed the different AS entities described in the literature [5]. Compared to HG patients, LFLG patients suffer from more comorbidities, exemplified by significantly higher STS scores. cLFLG patients are predominantly male and have the highest rates of CAD, prior MI and PCI and CKD. When examining echocardiographic characteristics with dilated atria and ventricles and reduced RV-function, they resemble patients with ischemic cardiomyopathy, which explains the low-flow state. In contrast, pLFLG patients are mainly female and have the highest frequency of AF and enlarged atria. A relevant fraction also has more than mild TR despite preserved right-ventricular function. These results are in accordance with the published literature [5].

The rate of atrioventricular valve regurgitation was higher among LFLG compared to HG patients, as expected. This is relevant because persistent MR or TR after TAVI is associated with a two-fold mortality increase, as recently reported by Winter et al. [19]. To what extent this is applicable particularly to LFLG patients remains to be elucidated. In these patients, subsequent interventional treatment of the atrio-ventricular valves in a timely manner after TAVI should be the subject of future studies.

### Technical outcomes are comparable

Concerning the composite endpoints according to VARC-3 [11], we found differences between the groups only in single components. The composite endpoints of technical failure and device failure were comparable. In paradoxical LFLG patients, bleeding rates and the need for vascular interventions were higher than in the other groups. This may be explained by the greater fraction of female patients with generally thinner blood vessels in this group. Classical LFLG patients have a higher 30-day mortality rate than the other two groups, which is not explained by procedural complications but may be due to underlying comorbidities.

### Mortality rates, predictors, and causes of death differ significantly

Mortality rates in both LFLG groups were higher after one, two, and three years, even after adjustment in comparison to HG, and mortality in classical LFLG was higher than in paradoxical LFLG. This result is in line with most published literature [8, 17] but in contrast to a meta-analysis from 2019 where mortality rates in pLFLG and cLFLG patients seemed comparable [20] and a propensity-matched analysis with comparable 1-year mortality in pLFLG and HG patients [10]. Interestingly, in our study, the death cause differed significantly between groups, and HG patients tended to have lower rates of cardiovascular death. According to Global Health Data Exchange, these rates are similar to the statistics on the cause of death in 70 to 89 year-old patients in Germany, which assumes a cardiovascular death in 40% [21]. Presumably, in most HG patients, AS was the key prognosis-limiting pathology whereas in LFLG patients, other cardiovascular comorbidities, too, have a strong influence on further clinical development.

Multivariate regression revealed that classical, but not paradoxical LFLG state was independently associated with an increased 3-year mortality rate. It indicates that paradoxical LFLG patients are comparable to HG patients to some extent. In the subset of patients with paradoxical LFLG, the higher rate of prognostically relevant comorbidities such as CKD or AF determines mortality. In this regard, our analysis is similar to the literature [8, 17]. Low-flow state has been described as an independent predictor of mortality in patients with preserved LVEF before [22]. However, whether this finding in these complex patients with numerous comorbidities might merely be a confounder remains questionable.

The high mortality rate among classical LFLG patients is only partly explained by other comorbidities, classical LFLG AS itself is an independent mortality predictor in our analysis. Classical LFLG patients are HFrEF patients and treating AS often only solves one of their problems, leaving exhausted ventricles, relevant atrioventricular valve regurgitations and arrhythmias behind.

### Gradients and NYHA functional class improve

Besides effects on mortality, symptomatic relief is equally important in this elderly patient population. Arguably, patients with relatively low dPmean at baseline may not profit to the same extent as HG patients. However, we could demonstrate, that gradients could be reduced significantly in all three groups. Our data also suggest that TAVI leads to subjective improvement in terms of NYHA functional class, irrespective of pre-TAVI gradients, which fits the current literature [23]. A reason for this might be the positive hemodynamic response to the relieved valve obstruction, that is especially important in patients with high filling pressures [24]. Furthermore, a recent analysis of the TOPAS trial concluded that aortic valve replacement in contrast to the best medical treatment would reduce mortality in cLFLG and pLFLG patients [25].

Diagnosis of severe AS in patients with mean pressure gradients below 40 mmHg is challenging and guidelines require different diagnostic modalities [1]. While the cause of low-flow is obvious in patients with reduced LVEF, it is less overt in paradoxical LFLG patients. In this real-world cohort of almost 2000 consecutive patients, half of which were in low-flow state, a significant reduction of symptoms could be achieved at an acceptable cost of complications, hinting at a sensible patient selection when considering aortic valve replacement. Future results from randomized prospective trials will further elucidate this issue and help select optimal treatment options. The optimal treatment for these patients has not yet been clearly defined and remains a case-to-case decision in the clinical practice and is subject of ongoing prospective and retrospective clinical trials (NCT03667365, NCT01835028, and NCT03863132).

### Limitations

This study is a single-center retrospective analysis with all its limitations. Due to selection bias, patients with expectedly low clinical improvement rates have not been treated with TAVI and were not part of this analysis; a comparison to patients only receiving the best medical treatment is not possible. Additionally, data on the medical therapy of the included patients were not available in enough detail for sufficient analysis.

The event rates of the analyzed endpoints, especially the single components of the composite endpoints, are low and need to be interpreted with caution. Another limitation is the incompleteness of the 3-year follow-up due to the inclusion of patients up until 2019. Adjustment of mortality analyses was only performed for differences in the STS score. Further, despite all echocardiography being reviewed in a core lab by an independent team, which is certainly a strength of this study, it was limited to the recorded images. Hence, data were not sufficient for the evaluation of diastolic dysfunction. Last, NYHA class is a subjective outcome parameter and not ideal to fully investigate clinical success.

## Conclusions

In this large-scale single-center analysis, LFLG AS is a relevant entity. TAVI was shown to be a safe treatment option for LFLG AS with similar complication rates compared to HG patients. While long-term mortality is high in these morbid patients, TAVI offers high potential for improvement of symptoms. Results of randomized studies to evaluate the current treatment algorithms, especially for paradoxical LFLG AS, will be of great interest.

## Supplementary Information

Below is the link to the electronic supplementary material.

Supplementary file1 (DOCX 348 KB)

## Abbreviations

AF: Atrial fibrillation
AKI: Acute kidney injury
AS: Aortic valve stenosis
CKD: Chronic kidney disease
dPmean: Mean pressure gradient
HG: High-gradient
LFLG: Low-flow low-gradient
LVEF: Left ventricular ejection fraction
PCI: Percutaneous coronary intervention
PVR: Paravalvular regurgitation
STS Score: The society of thoracic surgeons score
SVi: Stroke volume index
TAVI: Transcatheter aortic valve implantation
TR: Tricuspid regurgitation
VARC-3: Valve academic research consortium-3
