# Supplementary material for: TAVI in patients with low-flow low-gradient aortic stenosis–short-term and long-term outcomes
Source: Clin Res Cardiol. 2022 Mar 23;111(12):1325–35. doi: 10.1007/s00392-022-02011-4 (PMC9681695; doi:10.1007/s00392-022-02011-4)
Supplement: Supplementary file 1 — Supplementary file1 (DOCX 348 KB) [file 392_2022_2011_MOESM1_ESM.docx]

TAVI in patients with low-flow low-gradient aortic stenosis – short-term and long-term outcomes

# Figure titles and captions

## Supplemental Figure 1 Technical and clinical outcomes for all LFLG combined compared to HG patients


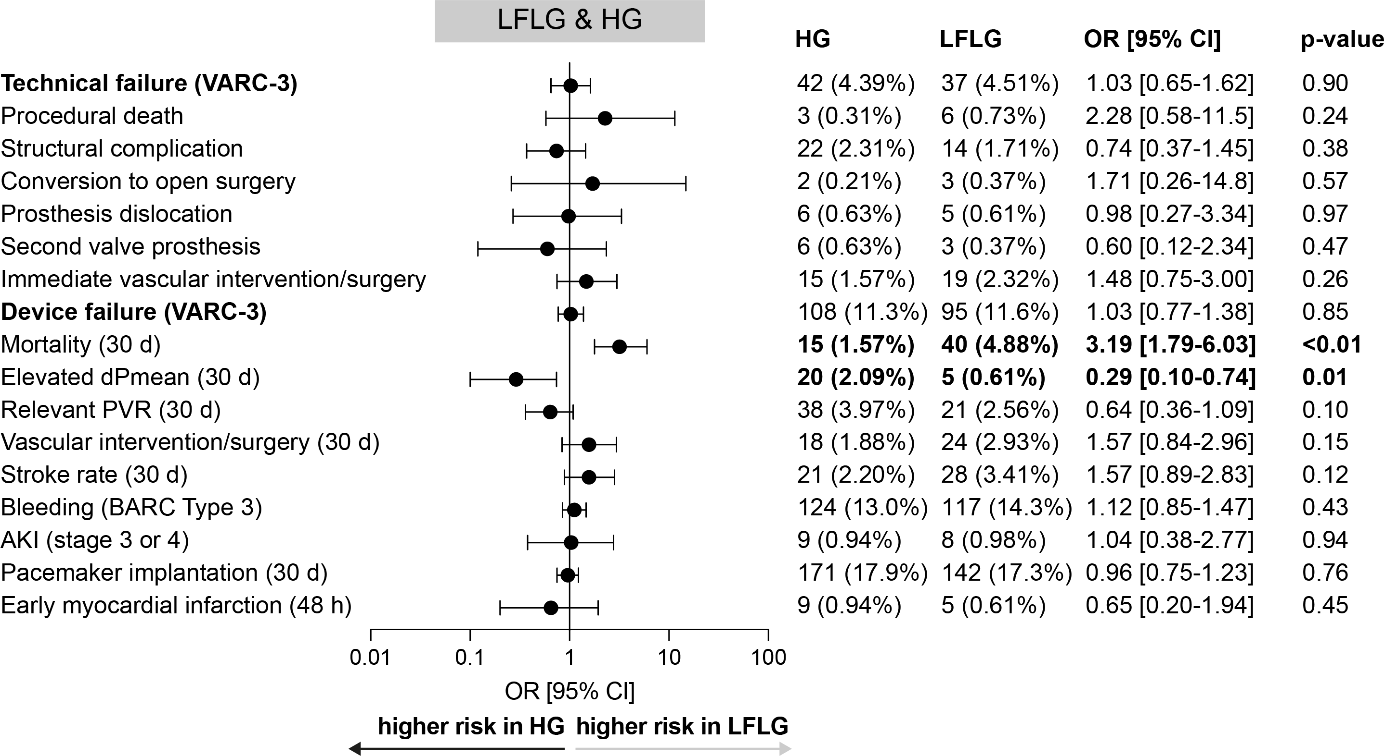
Short-term outcomes (up to 30 days) for all patients in the two LFLG groups combined were compared to HG according to Valve Academic Research Consortium-3 (VARC-3) endpoints. The composite endpoints of technical failure (consisting of procedural death, structural cardiac complications, conversion to open surgery, prosthesis dislocation, the use of a second valve prosthesis, or immediate vascular intervention or surgery) or device failure at 30 days (consisting of the composite endpoint technical failure, 30-day mortality, elevated pressure gradients or relevant paravalvular regurgitation on echocardiography, or vascular surgery/intervention at 30 days, stroke, relevant bleeding, acute kidney injury (AKI) and permanent pacemaker implantation) occurred at similar frequencies. However, there were differences in single components: 30-day mortality was higher in LFLG patients, and HG patients had a higher risk for elevated pressure gradients (dPmean) at 30 days.

## Supplemental Figure 2 Estimated 3-year mortality of all LFLG patients compared to HG patients


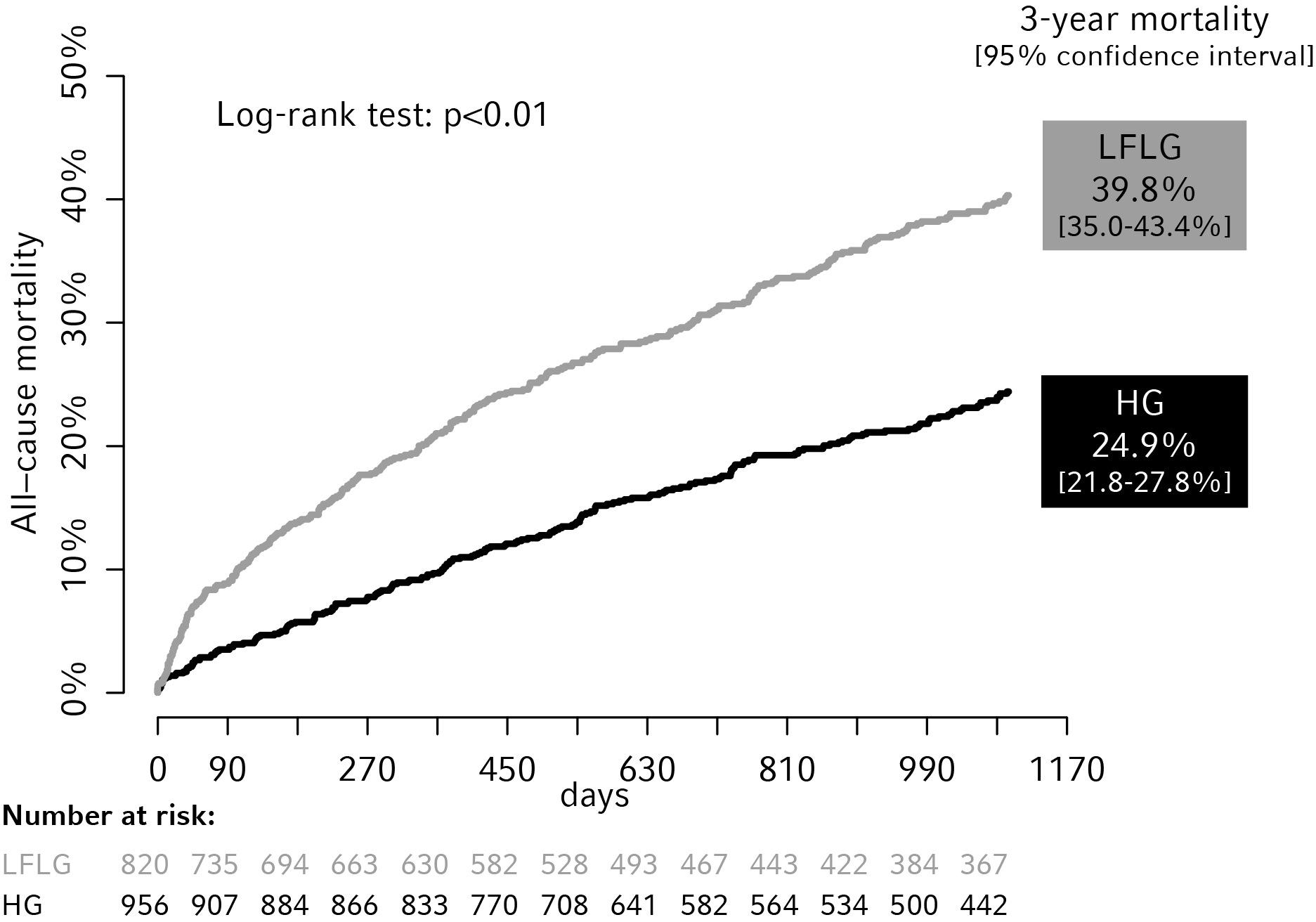


Kaplan-Meier curves depicting estimated 3-year mortality after TAVI of both LFLG groups combined in comparison to HG patients. Mortality rates were significantly different between groups at 1 year, 2 years and at 3 years. The hazard ratio for 3-year mortality for LFLG compared to HG is 1.8 [95% confidence interval, 1.5-2.2].

LFLG, low-flow low-gradient, HG, high gradient

# Supplemental tables

## Supplemental Table 1 Computer tomography measurements and procedural characteristics

|  | **HG  (N=956)** | **cLFLG (N=447)** | **pLFLG (N=373)** | **p value** |
| --- | --- | --- | --- | --- |
| Anulus area (cm^2^) | 4.5 [4.0-5.2] | 5.1 [4.5-5.8] | 4.3 [3.9-5.1] | **< 0.01** |
| Anulus perimeter (mm) | 77.8 [72.8-83.0] | 82.5 [76.5-87.8] | 76.1 [72.0-82.8] | **< 0.01** |
| **Prosthesis type** |  |  |  | 0.18 |
| Sapien | 777 (81.4%) | 378 (84.6%) | 291 (78.0%) |  |
| CoreValve | 61 (6.4%) | 23 (5.1%) | 31 (8.3%) |  |
| Other | 116 (12.2%) | 46 (10.3%) | 51 (13.7%) |  |
| **Prosthesis sizes** |  |  |  | **< 0.01** |
| <25 mm | 308 (32.4%) | 76 (17.1%) | 119 (32.2%) |  |
| 25-28 mm | 438 (46.1%) | 189 (42.5%) | 161 (43.6%) |  |
| >28 mm | 205 (21.6%) | 180 (40.4%) | 89 (24.1%) |  |
| Prosthesis size (mm) | 26.0 [23.0-26.0] | 26.0 [26.0-29.0] | 26.0 [23.0-27.0] | **< 0.01** |
| Predilatation | 764 (79.9%) | 314 (70.2%) | 266 (71.3%) | **< 0.01** |
| Postdilatation | 50 (5.2%) | 18 (4.0%) | 23 (6.2%) | 0.37 |
| Concomitant PCI | 107 (11.2%) | 81 (18.1%) | 48 (12.9%) | **< 0.01** |

Data are presented as n (%) or median [IQR]. A p-value of <0.05 was considered significant.

PCI, percutaneous coronary intervention

## Supplemental Table 2 Cause of death

|  | **HG** | **cLFLG** | **pLFLG** | **p value** |
| --- | --- | --- | --- | --- |
| **Cause of death** |  |  |  | **< 0.01** |
| Cardiovascular | 131 (46.0%) | 115 (55.6%) | 79 (56.4%) |  |
| “Advanced age” | 53 (18.6%) | 25 (12.1%) | 21 (15.0%) |  |
| Infection/sepsis | 30 (10.5%) | 28 (13.5%) | 11 (7.9%) |  |
| Cancer | 30 (10.5%) | 8 (3.9%) | 3 (2.1%) |  |
| Accident/suicide | 1 (0.4%) | 1 (0.5%) | 1 (0.7%) |  |
| Other | 40 (14.0%) | 30 (14.5%) | 25 (17.9%) |  |

Data are presented as n (%). A p-value of <0.05 was considered significant.

## Supplemental Table 3 Univariate analysis of predictors for 3-year mortality according to type of aortic stenosis

|  | HG | | cLFLG | | pLFLG | |
| --- | --- | --- | --- | --- | --- | --- |
| Variable | **HR [95% CI]** | **p value** | **HR [95% CI]** | **p value** | **HR [95% CI]** | **p value** |
| Male sex | 1.12 [0.85-1.47] | 0.42 | 1.07 [0.78-1.48] | 0.67 | 1.21 [0.84-1.75] | 0.31 |
| Age | **1.03 [1.01-1.06]** | **<0.01** | **1.03 [1.01-1.05]** | **0.01** | **1.05 [1.02-1.09]** | **<0.01** |
| BMI (kg/m^2^) | 0.97 [0.94-1.00] | 0.07 | 1.00 [0.97-1.03] | 0.96 | 0.98 [0.94-1.02] | 0.25 |
| STS score | **1.05 [1.04-1.07]** | **<0.01** | **1.06 [1.04-1.08]** | **<0.01** | **1.08 [1.05-1.11]** | **<0.01** |
| Diabetes | 0.87 [0.63-1.20] | 0.39 | 1.16 [0.85-1.58] | 0.36 | 1.20 [0.81-1.77] | 0.37 |
| Hypertension | 0.72 [0.47-1.08] | 0.11 | 1.69 [0.83-3.44] | 0.14 | 1.40 [0.62-3.20] | 0.42 |
| Smoking | 0.85 [0.59-1.24] | 0.41 | 0.95 [0.66-1.36] | 0.78 | 0.90 [0.53-1.54] | 0.71 |
| CKD | **1.92 [1.46-2.53]** | **<0.01** | **2.19 [1.56-3.07]** | **<0.01** | **1.66 [1.14-2.41]** | **0.01** |
| Atrial fibrillation | **1.74 [1.29-2.35]** | **<0.01** | 1.35 [1.01-1.82] | 0.05 | **1.51 [1.05-2.19]** | **0.03** |
| CAD | 1.27 [0.95-1.69] | 0.11 | **1.62 [1.11-2.35]** | **0.01** | 1.21 [0.81-1.81] | 0.36 |
| Prior MI | 1.25 [0.84-1.87] | 0.27 | 1.02 [0.73-1.42] | 0.90 | 1.31 [0.81-2.12] | 0.27 |
| Prior PCI | 1.05 [0.77-1.43] | 0.77 | 1.25 [0.92-1.68] | 0.15 | 1.29 [0.87-1.90] | 0.20 |
| AVA (cm^2^) | **0.27 [0.11-0.65]** | **<0.01** | 1.23 [0.51-2.97] | 0.64 | 1.85 [0.50-6.90] | 0.36 |
| dPmax (mmHg) | 1.01 [1.00-1.01] | 0.09 | **0.98 [0.97-0.99]** | **<0.01** | **0.98 [0.97-1.00]** | **0.04** |
| dPmean (mmHg) | 1.00 [0.99-1.02] | 0.66 | **0.96 [0.94-0.98]** | **<0.01** | **0.97 [0.94-0.99]** | **0.01** |
| SV (ml/m^2^) | **0.99 [0.98-1.00]** | **0.01** | **0.98 [0.97-1.00]** | **0.01** | 0.99 [0.97-1.01] | 0.18 |
| SVi (ml/m^2^) | 0.99 [0.97-1.00] | 0.05 | **0.97 [0.95-0.99]** | **0.01** | 0.98 [0.95-1.02] | 0.44 |
| LVEF (%) | **0.97 [0.95-0.98]** | **<0.01** | **0.96 [0.94-0.98]** | **<0.01** | **0.95 [0.91-1.00]** | **0.04** |
| TAPSE (mm) | **0.96 [0.93-0.99]** | **0.02** | **0.94 [0.91-0.97]** | **<0.01** | 0.99 [0.95-1.04] | 0.79 |
| AR grade 2 | 0.84 [0.52-1.37] | 0.48 | **0.47 [0.27-0.82]** | **0.01** | 1.05 [0.51-2.16] | 0.89 |
| MR>2 | 1.65 [0.92-2.95] | 0.09 | 1.48 [0.94-2.34] | 0.09 | 1.17 [0.48-2.87] | 0.73 |
| TR>1 | 1.26 [0.80-1.97] | 0.32 | **2.00 [1.43-2.80]** | **<0.01** | **2.08 [1.39-3.12]** | **<0.01** |
| RV/RA grad. (mmHg) | **1.02 [1.00-1.03]** | **0.01** | **1.01 [1.00-1.03]** | **0.03** | **1.02 [1.00-1.03]** | **0.02** |

BMI, body-mass index, STS-score, Society of Thoracic Surgeons score, CKD, chronic kidney disease, AF, atrial fibrillation, CAD, coronary artery disease, MI, myocardial infarction, PCI, percutaneous coronary intervention, AVA, aortic valve area, dPmax, maximum pressure gradient, dPmean, mean pressure gradient, SVi, stroke volume index, LVEF, left-ventricular ejection fraction, TAPSE, tricuspid annular plane systolic excursion, AR, aortic regurgitation, MR, mitral regurgitation, TR, tricuspid regurgitation, RV/RA grad., right-ventricular/right-atrial pressure gradient.

Data are presented as hazard ratio [95% confidence interval].

## Supplemental Table 4 Univariate and Multivariate Cox model for prediction of 3-year mortality in the entire study cohort

|  |  |  | Univariate | | Multivariate | |
| --- | --- | --- | --- | --- | --- | --- |
| Variable | **Living** | **Dead** | **HR [95% CI]** | **p value** | **HR [95% CI]** | **p value** |
| Group |  |  |  |  |  |  |
| HG | 752 (58.8%) | 204 (41.0%) | Ref. | Ref. |  |  |
| cLFLG | 266 (20.8%) | 181 (36.3%) | 2.16 [1.77-2.64] | **<0.01** | 1.73 [1.26-2.39] | **<0.01** |
| pLFLG | 260 (20.3%) | 113 (22.7%) | 1.53 [1.22-1.93] | **<0.01** | 1.21 [0.86-1.71] | 0.27 |
| Female sex | 641 (50.2%) | 218 (43.8%) | Ref. | Ref. |  |  |
| Male sex | 637 (49.8%) | 280 (56.2%) | 1.26 [1.06-1.51] | **0.01** | 1.16 [0.88-1.53] | 0.30 |
| Age (years) | 81.0 [77.4-85.1] | 83.5 [78.2-87.3] | 1.04 [1.02-1.05] | **<0.01** | 1.03 [1.01-1.06] | **<0.01** |
| BMI (kg/m^2^) | 26.0 [23.6-29.3] | 25.3 [22.5-28.7] | 0.98 [0.96-1.00] | **0.04** | 0.98 [0.95-1.01] | 0.15 |
| Diabetes | 3.00 [2.00-5.00] | 5.09 [3.04-8.13] | 1.06 [1.05-1.07] | 0.340 |  |  |
| Hypertension | 360 (30.3%) | 155 (33.6%) | 1.09 [0.90-1.32] | 0.65 |  |  |
| Smoking | 1075 (90.3%) | 426 (91.4%) | 1.08 [0.78-1.49] | 0.54 |  |  |
| CKD | 243 (20.4%) | 88 (18.9%) | 0.93 [0.74-1.17] | **<0.01** | 1.83 [1.39-2.41] | **<0.01** |
| Atrial fibrillation | 509 (39.8%) | 315 (63.3%) | 2.13 [1.77-2.55] | **<0.01** | 1.31 [0.99-1.73] | 0.06 |
| CAD | 319 (25.0%) | 192 (38.6%) | 1.69 [1.41-2.02] | **<0.01** | 1.34 [0.98-1.84] | 0.07 |
| Prior MI | 705 (59.1%) | 331 (70.0%) | 1.48 [1.21-1.80] | **0.01** | 0.93 [0.65-1.33] | 0.68 |
| Prior PCI | 180 (14.1%) | 96 (19.3%) | 1.33 [1.06-1.66] | **0.01** | 0.91 [0.65-1.25] | 0.55 |
| TAPSE (mm) | 20.0 [17.0-24.0] | 19.0 [15.0-22.0] | 0.94 [0.92-0.96] | **<0.01** | 1.00 [0.97-1.03] | 0.86 |
| AR grade 2 | 140 (11.0%) | 39 (7.83%) | 0.73 [0.52-1.01] | 0.06 |  |  |
| MR>2 | 55 (4.32%) | 38 (7.65%) | 1.67 [1.20-2.32] | **0.01** | 0.99 [0.65-1.51] | 0.96 |
| TR>1 | 134 (11.1%) | 102 (21.4%) | 1.95 [1.57-2.43] | **<0.01** | 1.26 [0.93-1.71] | 0.13 |
| RV/RA grad. (mmHg) | 34.0 [26.0-44.0] | 38.0 [29.0-48.0] | 1.02 [1.01-1.02] | **<0.01** | 1.01 [1.00-1.02] | **0.03** |

HG, high gradient aortic stenosis, cLFLG, classical low-flow low-gradient aortic stenosis, pLFLG, paradoxical low-flow low-gradient aortic stenosis, BMI, body-mass index, STS-score, Society of Thoracic Surgeons score, CKD, chronic kidney disease, AF, atrial fibrillation, CAD, coronary artery disease, MI, myocardial infarction, PCI, percutaneous coronary intervention, AVA, aortic valve area, TAPSE, tricuspid annular plane systolic excursion, AR, aortic regurgitation, MR, mitral regurgitation, TR, tricuspid regurgitation, RV/RA grad., right-ventricular/right atrial-pressure gradient.

Data are presented as hazard ratio (HR) [95% confidence interval
